# A case-control study on depression, anxiety, and belief in sexual myths in trans women

**DOI:** 10.3389/fpsyt.2022.955577

**Published:** 2023-01-09

**Authors:** Betul Uyar, Ilyas Yucel, Emre Uyar, Elif Ateş Budak, Ilker Kelle, Semra Bulbuloglu

**Affiliations:** ^1^Department of Psychiatry, Faculty of Medicine, Dicle University, Diyarbakir, Turkey; ^2^Department of Medical Biology and Genetics, Faculty of Medicine, Dicle University, Diyarbakir, Turkey; ^3^Department of Pharmacology, Faculty of Pharmacy, Inonu University, Malatya, Turkey; ^4^Department of Medical Pharmacology, Faculty of Medicine, Dicle University, Diyarbakir, Turkey; ^5^Division of Surgical Nursing, Nursing Department, Faculty of Health Sciences, Istanbul Aydin University, Istanbul, Turkey

**Keywords:** anxiety, belief in sexual myths, depression, gender dysphoria, sexual myths, trans women

## Abstract

**Objective:**

The purpose of our study was to investigate depression, anxiety, and belief in sexual myths in trans women.

**Methods:**

This is a prospective case-control study. The case group included 60 trans women who were referred to the Medical Biology and Genetics Department from various clinics of the research and training hospital where this study was conducted. The control group consisted of 60 healthy male individuals who presented to the same hospital for routine health follow-ups and collecting documents showing their health. In data collection, we used a Personal Information Form, the Sexual Myths Scale, and the Beck Depression and Anxiety Inventories. The IBM Statistical Package for the Social Sciences 25.0 was used to analyze the data.

**Results:**

In the case group, 26.7% of the participants were sex workers, and all were single. While 46.7% of the participants in the case group were living with their families, 66.7% were smokers, and 13.3% were receiving hormone treatment. All 60 participants in the control group were also single. The participants in the control group had higher levels of believing sexual myths and lower levels of anxiety and depression than those in the case group (*p* = 0.000). The mean scores of the participants in the control group in the Sexual Orientation and Sexual Violence subscales of the Sexual Myths Scale were higher than the mean scores of those in the case group (*p* < 0.05).

**Conclusion:**

The trans women who participated in this study had higher levels of anxiety and depression and lower levels of believing sexual myths than the control group. The mental health of trans women can be disrupted due to various treatments they are exposed to in society such as stigma, discrimination, and violence. Their higher anxiety and depression levels in this study could be explained by this exposure. This exposure could also have led to their lower total scores in the Sexual Myths Scale, as well as lower scores in the Sexual Violence and Sexual Orientation subscales.

## Introduction

Gender incongruence is described as “an individual experiences a marked and persistent mismatch between the gender they experience and their assigned gender” (1, 2). People with gender incongruence experience a mismatch between their assigned gender and gender identity. Based on this mismatch, the person may want to have surgical interventions and hormone treatments. They wish to change their name in social life, have the social status of the opposite sex, behave like the opposite sex, and change their outer appearance. The ICD-11 diagnostic system defines these individuals as “transgender” (3). DSM-5 reported the prevalence of being gender dysphoria as 0.005–0.014% in male-to-female individuals and 0.002–0.003% in female-to-male individuals. In the Diagnostic and Statistical Manual of Mental Disorders, Fifth Edition (DSM-5), Gender incongruence is defined as the presence of a significant discrepancy between one’s existing gender identity and their assigned gender for at least 6 months (4).

In Türkiye, according to the Turkish Civil Code 4721, Part Two and 40th Item, a person who wants to change their sex can officially demand sex change by applying to a court in person. For this application, the court considers the requirements for approval as that the person making the application is over the age of 18 years, they are not married, and they can document with an official health committee report to be obtained from a research and training hospital that they are transsexual, their sex change is imperative for their mental health (5).

The term trans woman refers to individuals who are born with male biology but identify as the female sex (6). It is not possible to argue that a complete sexual transition occurs even for trans women who have undergone surgical interventions and hormone treatments. In Türkiye, trans women are often exposed to physical and verbal violence, experience unemployment and lack of money, and are abandoned by a heterosexual partner when they start the gender transition process (7). Trans women are excluded from society. This situation paves the way for the development of anxiety and depression in trans women (8–10). Anxiety is one of the most frequently encountered disorders worldwide (11), it interrupts activities of daily living and reduces quality of life to a substantial degree (12). Anxiety disorders may result in the avoidance of social life and many social disorders (13). It is believed that by 2030, depression will be the leading cause of disease burden in developed countries (14). It was reported that as the severity of depression increases, the prevalence of individual dysfunctions also increases (15).

Sexuality is a complex and dynamic process that exists in life from childhood to old age (16). Sexual health is associated with both physical and mental well-being (17). The meaning attributed to sexuality and the concept of sexual health is significantly influenced by religious rules, prejudices, taboos, traditions, and customs. Beliefs that do not have a scientific foundation, are related to the inaccurate beliefs and thoughts of individuals, connected to superstitions that have existed in society since ancient times, and accepted as true, while not having any scientific connection with sexuality are defined as “sexual myths” (18, 19). Sexual myths may lead to sexual dysfunctions, gender identity disorder, and lowered quality of sexual life (20, 21).

Stigma, discrimination, violence, and mistreatment in childhood may play an effective role in the deterioration of the mental health of trans women. Many trans women may experience discrimination at varying degrees in terms of access to housing support, healthcare, employment, education, social assistance, and many other social services (22). It was reported that the stress of stigma and trying to cope with this stress cause barriers to the acceptance of the gender identity of trans women and their increased vulnerability in this process (23). This situation may lead trans individuals toward depression and anxiety. The literature review that was carried out for this study did not reveal any previous study that examined whether beliefs in sexual myths are higher among individuals who live in conformity with society and are traditionalists or trans women. Sexual myths may be an obstacle for trans women to freely experience their gender preference. The hypothesis of this study is that anxiety and depression may be in trans women and this negative mental state is worsened by belief in sexual myths. In our study, we aimed to investigate depression, anxiety, and belief in sexual myths in trans women with a case-control design.

## Materials and methods

### Design and participants

This is a case-control study and the data of the study were collected prospectively. The population of the study consisted of 76 in trans women who presented to the Endocrinology, Urology, Psychiatry, and Obstetrics clinics of the research and training hospital where this study was conducted for sex change and referred to the Medical Biology and Genetics Department of the same hospital. The sample size was calculated using the G*Power 3.1.9.7 software. According to this program, a total of 120 participants were divided into experimental and control groups with 0.5 effect size, 0.05 margin of error, 0.95 confidence level and 95% confidence interval, and thus each group included 60. Researchers could not reach three trans women from their registered phone numbers, five trans women could not participate because they resided outside the city, and eight trans women did not voluntarily participate in the study. A total of 16 trans women were excluded from the study. The control group consisted of 60 healthy male individuals who presented to the same hospital for routine health follow-ups and collecting documents showing their health. The total number of participants in our study was 120. We used the purposive sampling method to include the participants. The inclusion and exclusion criteria for the study were as follows.

### Inclusion criteria

i. Voluntarily agreeing to participate in the study.
ii. The criterion for care at the hospital was to have gender dysphoria.
iii. Individuals who will form both samples must be between the ages of 18 and 65.
iv. Not having a history of or being under treatment for a life-threatening, severe disease.
v. Not having a diagnosis or history of diagnosis of a psychiatric condition that would cause substantial mental infirmity (Schizophrenia, delirium, cognitive problems etc.).

### Exclusion criteria

i. Being illiterate
ii. Being unable to speak Turkish
iii. Having a history of or being under treatment for a life-threatening, severe disease
iv. Disorders of sex development
v. Being under 18 or over 65 and not male

In the hospital where the study was conducted, the diagnosis of Gender Dysphoria is determined based on the DSM-5 diagnostic criteria. Processes are initiated to ensure that individuals diagnosed with gender dysphoria according to DSM-5 enter the process of gender transition. Individuals in the control group were individuals who applied to the hospital where the study was conducted to obtain a health report due to work and education. They wanted to go through a routine physician examination for a general medical examination, measurement of blood values, and health check-up. Control group participants who met the study’s inclusion criteria were included in the sample after obtaining informed consent.

### Data collection

The data of this study were collected by the researchers using the face-to-face interview and survey methods between November 2019 and December 2021. After the case and control groups were formed, the researchers met each participant individually, and all participants provided informed consent. Afterward, the data collection instruments were given to each participant, and each participant was asked to fill them out within approximately 15 min.

### Data collection instruments

In data collection, we used a Personal Information Form, the Sexual Myths Scale, and the Beck Depression and Anxiety Inventories.

### Personal information form

This form included questions on the sociodemographic characteristics of the participants including age, marital status, income status, and education status.

### Sexual myths scale

Gölbaşı et al. developed the Sexual Myths Scale (SMS) and tested its validity and reliability (24). It is a 5-point Likert-type scale consisting of 28 items that is used to identify the levels of belief in sexual myths in individuals. The scale has eight subscales. These subscales are Sexual Orientation: items 1–5, Gender: items 6–11, Age and Sexuality: items 12–15, Sexual Behavior: items 16–18, Masturbation: items 19–20, Sexual Violence: items 21–24, Sexual Intercourse: items 25–26, and Sexual Satisfaction: items 27–28. Each item has the response options of Absolutely Disagree (i), Disagree (ii), Undecided (iii), Agree (iv), and Absolutely Agree (v). The total score of the Sexual Myths Scale is found by summing the scores of all items in the scale, while the score of each subscale is calculated by summing the scores of the items in that subscale. The scale does not have a cutoff point, and higher scores indicate higher levels of belief in sexual myths. The Cronbach’s alpha internal consistency coefficient of the scale was reported as 0.91 initially and 0.81 in the repeated reliability test (24). In this study, the Cronbach’s alpha coefficient of the scale was found as 0.94.

### Beck depression inventory (BDI)

Beck Depression Inventory (BDI) was developed in 1961 by Beck et al. (25) to measure the severity of behavioral symptoms of depression and monitor changes with treatment. In BDI, depression-specific behaviors and symptoms are defined, and each statement is scored in the range of 0–3. It consists of 21 items with response options ordered from the mildest to the most severe. The patient is asked to mark the statements defining their current condition best, and the total score is calculated by summing the scores of all items. The severity of depression is defined based on the scores as 0–9 = minimal, 10–16 = mild, 17–29 = moderate, and 30–63 = severe. The subscale scores are calculated using the cognitive-affective factor and the somatic performance factor. The scale was translated into Turkish and tested for validity and reliability in Turkish by Hisli (26). In this study, the Cronbach’s alpha coefficient of the scale was calculated as 0.91.

### Beck anxiety inventory (BAI)

BAI measures the frequency of anxiety symptoms experienced by the individual. It is a 4-point Likert-type self-assessment scale, and each item is scored in the range of 0–3. Higher total scores indicate higher severity of anxiety experienced by the individual. It was developed by Beck et al. (27) and tested for validity and reliability in Türkiye by Ulusoy et al. (28). The severity of anxiety is defined based on the scores as 8–15 = mild anxiety, 16–25 = moderate anxiety, and 26–63 = severe anxiety. In this study, the Cronbach’s alpha coefficient of the scale was calculated as 0.89.

### Statistical analysis

The data were coded by the researchers and analyzed using the IBM Statistical Package for the Social Sciences (SPSS) 25.0. Descriptive statistics were calculated. The homogeneity test between the case and control groups was conducted using Chi-squared test and analysis of variance (ANOVA). The normality of the distribution of the data was tested using Kolmogorov-Smirnov test before starting the statistical analyses and the groups did not show a normal distribution within themselves. Independent-samples *t*-test was used to determine the relationships between the scales. The results were interpreted in a 95% confidence interval and a statistical significance level of *p* < 0.05.

### Ethical aspect of the study

Before our study, approval was obtained from Dicle University Faculty of Medicine Non-Invasive Clinical Research Ethics Committee (Date: 02/10/2019, Number: 123). In line with the principles of the Declaration of Helsinki, the participants in the case and control groups were informed about the study, and the participants signed the Informed Consent Form. The individuals who voluntarily agreed to participate were included after they provided verbal and written consent.

## Results

Table 1 presents the descriptive characteristics of the case and control groups, and the homogeneity test results. All 60 trans women in the case group were single. While 23.3% of the participants in the case group were university students, laborers had the same rate, and 26.7% were sex workers. In the case group, 46.7% of the participants were living with their families, 66.7% were smokers, and 13.3% were receiving hormone treatment. All 60 men in the control group were also single. While 53.3% of the participants in the control group were high school graduates, 26.7% were laborers. The tests in our study showed that the groups were homogeneously distributed.

**TABLE 1:** Descriptive characteristics of case and control groups and homogeneity test results (*n* = 120).

Characteristics	Case group (*n* = 60)		Control group (*n* = 60)		Homogeneity test between groups
**Age (years, X ± SD)**	**24.5 ± 3.19 (min: 20, max: 35)**		**24.6 ± 4.07 (min: 18, max: 33)**		**F = 0.751** ***p* = 0.744**
	***n***	**%**	***n***	**%**	
**Marital status**					
Single	60	100	60	100	χ^2^ = 1.018 *p* = 0.903
Married	–	–	–	–	
**Education status**					
Literate with no formal degree	4	6.7	2	3.3	χ^2^ = 0.312 *p* = 0.515
Primary school	2	3.3	8	13.3	
High school	34	56.7	32	53.3	
University or higher	20	33.3	18	30	
**Occupation**					
Civil servant	6	10.0	10	16.7	χ^2^ = 1.721 *p* = 0.313
Laborer	14	23.3	16	26.7	
Freelance	10	16.7	10	16.7	
Student	14	23.3	24	40	
Sex worker	16	26.7	–	–	
**Income status**					
Income < expenses	24	40	18	30	χ^2^ = 1.817 *p* = 0.628
Income = expenses	16	26.7	16	26.7	
Income > expenses	10	16.7	6	10	
Income much higher than expenses	10	16.7	6	10	
**Living with**					
With family (e.g., mother, father)	28	46.7	46	76.7	χ^2^ = 0.813 *p* = 0.514
Alone	20	33.3	6	10	
With friends	12	20	8	13.3	
**Smoker**					
Yes	40	66.7	34	56.7	χ^2^ = 1.212 *p* = 0.216
No	20	33.3	26	43.3	
**Consumes alcohol**					
Yes	24	40	21	35	χ^2^ = 1.618 *p* = 0.416
No	36	60	39	65	
**Uses addictive substances such as marijuana and heroin**					
Yes	8	13.3	2	3.3	χ2 = 1.148 *p* = 0.544
No	52	86.7	58	96.7	
**Receiving hormone treatment**					
Yes	8	13.3	–	–	–
No	52	86.7	60	100	

$\bar{x}$, mean; SD, standard deviation; F, ANOVA, one-way analysis of variance; χ^2^, Chi-squared test.

The mean SMS, BDI, and BAI scores of the participants in the case and control groups are presented in Table 2.

**TABLE 2:** Mean sexual myths scale (SMS), Beck depression inventory (BDI), and Beck anxiety inventory (BAI) scores of case and control groups (*N* = 120).

Scales	Items	Score range	Case group (*n* = 60)		Control group (*n* = 60)	
		**Min.-Max.**	**$\bar{\text{x}}$** **+** **SD**	**Min.-Max.**	**$\bar{\text{x}}$** **+** **SD**	**Min.-Max.**
Sexual myths scale	1–28	28–140	68.9 ± 13.8	39–97	82.6 ± 18.29	45–110
Sexual orientation	1–5	5–25	10.7 ± 4.6	6–20	14.3 ± 5.35	6–24
Gender	6–11	6–55	16.06 ± 5.28	6–25	16.8 ± 6.15	6–26
Age and sexuality	12–15	4–20	11.7 ± 3.87	4–17	12.7 ± 3.72	4–17
Sexual behavior	16–18	3–15	9.3 ± 2.39	4–13	10.96 ± 2.23	5–14
Masturbation	19,20	2–10	7.4 ± 1.99	2–9	7.8 ± 1.71	4–10
Sexual violence	21–24	4–20	7.26 ± 1.83	4–11	9.66 ± 3.78	5–17
Sexual intercourse	25,26	2–10	4.03 ± 1.56	2–7	4.96 ± 2.37	2–9
Sexual satisfaction	27,28	2,10	2.4 ± 1.27	2–7	5.46 ± 3.1	2–9
Beck depression inventory	1–21	0–63	12.97 ± 6.65	0–22	10.93 ± 4.15	5–20
Beck anxiety inventory	1–21	0–63	15.4 ± 7.04	0–51	10.13 ± 4.82	4–21

As seen in Table 2, the mean SMS score of the case group was 68.9 ± 13.8 (min: 39, max: 97), while the mean SMS score of the control group was 82.6 ± 18.29 (min: 45, max: 110). The mean BDI scores were 12.97 ± 6.65 (min: 0, max: 22) in the case group and 10.93 ± 4.15 (min: 5, max: 20) in the control group. Moreover, the mean BAI scores were 15.4 ± 7.04 (min: 0, max: 51) in the case group and 10.13 ± 4.82 (min: 4, max: 21) in the control group.

Table 3 shows the results of the comparisons of the levels of depression, anxiety, and believing sexual myths in the case and control groups.

**TABLE 3:** Comparisons of levels of depression, anxiety, and believing sexual myths in case and control groups (*N* = 120).

Scales	Case group (*n* = 60)	Control group (n = 60)	Intergroup comparison
	**$\bar{\text{x}}$** **+** **SD**	**$\bar{\text{x}}$+** **SD**	**Test and significance**
Sexual myths scale	68.9 ± 13.8	82.6 ± 18.29	*t* = 0.632 *p* = 0.000**
Sexual orientation	10.7 ± 4.6	14.3 ± 5.35	*t* = 1.284 *p* = 0.000**
Gender	16.06 ± 5.28	16.8 ± 6.15	*t* = 1.324
Age and sexuality	11.7 ± 3.87	12.7 ± 3.72	*p* = 0.413
Sexual behavior	9.3 ± 2.39	10.96 ± 2.23	*t* = 1.211 *p* = 0.631
Masturbation	7.4 ± 1.99	7.8 ± 1.71	*t* = 0.417 *p* = 0.073
Sexual violence	7.26 ± 1.83	9.66 ± 3.78	*t* = 1.107 *p* = 0.003**
Sexual intercourse	4.03 ± 1.56	4.96 ± 2.37	*t* = 0.544 *p* = 0.077
Sexual satisfaction	2.4 ± 1.27	5.46 ± 3.1	*t* = 3.154 *p* = 0.015*
Beck depression inventory	12.97 ± 6.65	10.93 ± 4.15	*t* = 0.147 *p* = 0.004**
Beck anxiety inventory	15.4 ± 7.04	10.13 ± 4.82	*t* = 1.457 *p* = 0.000**

$\bar{x}$, mean; SD, standard deviation; t, Independent-samples t-test. **p < 0.01, *p < 0.05.

As shown in Table 3, the control group had higher levels of believing sexual myths and lower levels of depression and anxiety in comparison to the case group (*p* = 0.000). The control group had higher mean scores in all subscales of SMS, while these differences were statistically significant for the Sexual Orientation and Sexual Violence subscales (*p* < 0.05).

**FIGURE 1:**
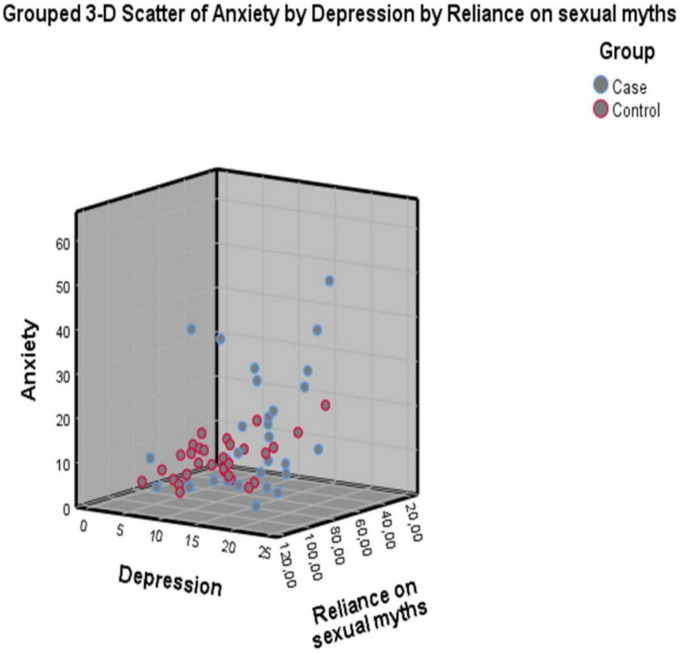
Scatter plot of believing sexual myths, depression, and anxiety in case and control groups (*N* = 120).

Figure 1 clearly demonstrates the higher anxiety and depression levels and lower levels of believing sexual myths in the case group. Moreover, the lower anxiety and depression levels and higher levels of believing sexual myths in the control group can be seen in the Figure 1.

## Discussion

We examined the level of depression, anxiety and belief in sexual myths of trans women in a case and controlled manner in the study. The hypothesis of this study was that in addition to the difficulties of being a trans woman, belief in sexual myths triggered anxiety and depression. In this study, we found that the trans women in the case group had anxiety and depression, whereas the participants in the control group had significantly lower anxiety and depression scores than those in the case group (*p* < 0.01). The identity and behaviors of trans women are usually stigmatized in society, and this leads them to become at risk of negative health outcomes. In many societies, trans women are exposed to high rates of discrimination and violence regarding their sexual identity (29). Studies comparing trans women to their siblings or the general public have reported that trans women are exposed to higher levels of discrimination (30). A study that was conducted in Türkiye revealed that trans women faced stigma and various forms of discrimination in many areas of social life (31). Previous studies have found a higher prevalence of mental disorders, especially major depression and anxiety, among trans women compared to the general public (32, 33). Major depression and anxiety disorders were encountered at high rates in trans women, and it was determined that 38% of these individuals were diagnosed with mental disorders, and their lifelong prevalence of mental disorders was 70% (34). The lifelong prevalence of mental disorders among trans women in Türkiye has been reported similar to those in the international literature (35, 36). Two different studies reported the rate of depression in trans women as 40% (33) and 51% (37). Anxiety rates in trans women have been found between 26 and 40% (37–39). Previous studies have focused on the prevalence of depression and anxiety. Our study examined the levels of depression and anxiety in individuals, and thus, it is an important source of data.

In our study, the rates of believing sexual myths in the control group were higher than those in the case group. A study that was carried out in Türkiye with the participation of 167 men revealed a high rate of believing sexual myths (40). It is clear that sexual myths brought about by the social structure of Türkiye that includes patriarchal and traditionalist approaches create pressure on the expression and experience of the sexual identity among trans women. Nevertheless, the results of studies in other countries, including Western societies, have also shown that the areas of freedom enjoyed by trans women are limited, and this situation leads to anxiety and depression, as well as various other mental issues. Incomplete or inaccurate knowledge of sexuality acquired in the psychosexual development stage in childhood and adolescence, and inaccurate and exaggerated expectations about sexuality are reflected in the sexual beliefs and behaviors of the individual in their adulthood, and they pave the way for problems to be experienced in their sexual life. This issue may explain the higher levels of believing sexual myths among the participants in the control group than those in the case group.

The control group in this study had significantly higher total Sexual Myths Scale (SMS) scores and scores in the Sexual Orientation and Sexual Violence subscales of SMS than the case group (*p* < 0.05). This result showed that the levels of belief in sexual myths among the trans women were lower compared to the men without gender dysphoria. To the best of our knowledge, there is no study investigating belief in sexual myths among trans women in the literature. The possibility that trans women may have different sexual experiences or witness such experiences in periods when they are in a search regarding their sexual identity could lead them to become more open-minded about sexuality. Moreover, this possibility may also set a basis for their higher levels of exposure to sexual violence, discrimination, and stigma in comparison to the general population. This exposure may have led to lower levels of believing sexual myths in trans individuals than in the general population in more traditional societies.

In this study, trans women had low levels of belief in sexual myths in addition to high levels of anxiety and depression. Individuals in the control group had lower levels of anxiety and depression and higher levels of belief in sexual myths than trans women. A previous study conducted with the participation of women and men in the premarital period, the level of belief in sexual myths of individuals who are experienced in sexual intercourse and who have sufficient knowledge about sexuality was found to be lower than those who are inexperienced and less knowledgeable (41). Other studies have reported that as sexual experience dwindles, sexual myths decrease, with men believing more in sexual myths than women (18, 19). Beliefs are influenced by experiences. In our study, we did not examine the sexual experience and knowledge levels of the sample groups. It is not possible for us to comment on which group is more experienced and knowledgeable from our findings. Trans women may have developed a tolerance for different sexual experiences while seeking sexual identity and may therefore tend to be more open-minded about their sexuality. Our results can form the basis for future studies on belief in sexual myths in trans women.

Unfortunately, although we are in the 21st century, Turkish society has not become adequately free about different sexual identities. Individuals who have not accepted that gender is not limited to a binary system, it is a construct that covers a broad spectrum of sexual identities, and the gender of a person can be different than their biological sex cannot be expected to be open-minded about sexuality or any other issue. If society becomes more tolerant and open-minded about these issues, it will be possible to create a more pleasant life for individuals with differences. This way, individuals in society can get over their sexual attitudes and knowledge based on taboos, namely sexual myths. Furthermore, it will be possible to provide everyone with healthier ways of socializing, sexual preferences suitable for their individual identity, and a freer and more respectful social life.

### Limitations

This study, in which we investigated trans women’s belief in anxiety, depression, and sexual myths, has several limitations. We can list these limitations as cross-sectional design, single-center study, and uncertain generalizability of the results.

## Conclusion

The trans women who participated in this study had higher levels of anxiety and depression than the control group, and this result was compatible with those in the literature. Factors such as the exposure of trans women to stigma, discrimination, and violence may be associated with their higher levels of depression and anxiety. On the other hand, they had lower levels of believing sexual myths compared to the control group. This result may be connected to different sexual experiences of trans women or their witnessing of such experiences in periods when they are in a search regarding their sexual identity, and their possibility of being more open-minded about sexuality due to these factors. Additionally, their exposure to mistreatment by society could explain their lower scores in the Sexual Orientation and Sexual Violence subscales of the Sexual Myths Scale. Awareness can be raised in society by educating people about different genders and gender identities. This way, an environment that is mentally healthier, freer, more respectful, and more tolerant can be created for everyone.

## Data availability statement

The original contributions presented in this study are included in the article/supplementary material, further inquiries can be directed to the corresponding author.

## Ethics statement

The studies involving human participants were reviewed and approved by Dicle University Faculty of Medicine Non-Invasive Clinical Research Ethics Committee (Date: 02/10/2019, Number: 123). The patients/participants provided their written informed consent to participate in this study.

## Author contributions

BU, IY, EU, EA, IK, and SB: medical practices, design, and writing. BU, IY, and EU: concept. BU and EU: data collection or processing. BU and IK: analysis or interpretation. SB, BU, and IY: literature search. All authors contributed to the article and approved the submitted version.
